# Dysregulated Non-Coding RNA Expression in T Cells from Patients with Ankylosing Spondylitis Contributes to Its Immunopathogenesis

**DOI:** 10.3390/biomedicines12081873

**Published:** 2024-08-16

**Authors:** Hui-Chun Yu, Sz-Tsan Wang, Ming-Chi Lu

**Affiliations:** 1 Division of Allergy, Immunology and Rheumatology, Dalin Tzu Chi Hospital, Buddhist Tzu Chi Medical Foundation, Chiayi 622401, Taiwan; 2 School of Medicine, Tzu Chi University, Hualien 970374, Taiwan

**Keywords:** ankylosing spondylitis, T cells, non-coding RNAs, immunopathogenesis, cytokines, micro RNAs

## Abstract

Ankylosing spondylitis (AS) is a chronic inflammatory disorder characterized by inflammatory back pain and bony fusion of vertebral joints. Genetic associations and environmental factors have been proposed to explain the immunopathogenesis of AS. In the past few years, there have been major advances in understanding T cell dysfunction in AS. Clinically, targeting interleukin-17A, a major cytokine secreted by T helper 17 cells, has been approved for treating patients with active AS. Non-coding RNAs (ncRNAs) are RNA transcripts that do not translate into proteins. The ncRNAs regulate both innate and adaptive immunity and participate in the pathogenesis of autoimmune diseases, including AS. The main purpose of this article is to review the up-to-date studies investigating the aberrant expression of ncRNAs in T cells from patients with AS and to summarize their roles in its pathogenesis. After searching PubMed for studies published between January 2013 and June 2024, nine studies investigating the expression of ncRNAs in AS T cells were included. We found that aberrantly expressed ncRNAs in AS T cells could cause abnormal cytokine release, cell signaling abnormalities, and dysregulated cell proliferation and death, which contribute to the immunopathogenesis of AS. We discussed some limitations of these studies and suggested several research fields for further investigation.

## 1. Introduction

Ankylosing spondylitis (AS), currently named radiographic axial spondyloarthritis (r-axSpA), is an inflammatory disease characterized by inflammatory back pain [1]. Chronic inflammation of AS might lead to the bony fusion of vertebral joints, resulting in the disability of the patients. In addition to spinal involvement, patients with AS may also suffer from enthesis, peripheral arthritis, and extra-articular manifestations, including psoriasis, uveitis, and inflammatory bowel disease [2]. The prevalence of AS ranges from approximately 0.32% to 1.4%, with a male-to-female ratio of around 2–3:1 [3]. Patients with AS have increased mortality, especially from cardiovascular disease [4].

The pathogenesis of AS is complex, involving both innate and adaptive immune responses. Genetic associations, particularly human leukocyte antigen (HLA)-B27, have drawn major attention [5]. In recent years, genomics and proteomics advancement have changed our knowledge on the pathogenesis and diagnosis for many diseases [6]. In addition, there have been major advances in understanding the critical role of T cells in the immunopathogenesis of AS [7]. Currently, it is known that T-cell function is regulated not only by gene expression and protein production but also by epigenetic changes (DNA methylation, histone modification, and ncRNAs) and contributes to T cell dysfunction in autoimmune diseases [8]. It is not surprising that abnormal expression of ncRNAs in T cells from patients with AS could contribute to its pathogenesis. Moreover, ncRNAs is a rapidly growing research field. Therefore, the main purpose of this article is to review the critical role of aberrant expression of non-coding RNAs (ncRNAs) in T cells from patients with AS and to explore their potential role in its pathogenesis. Since the studies included in this manuscript still use the term ankylosing spondylitis, we will use this term throughout the article. We searched PubMed and used the terms: “ankylosing spondylitis or axial spondyloarthritis”, “ncRNAs”, and “T cells” for studies published from January 2013 to June 2024. Twenty papers were found, and after excluding review articles and those not relevant to the expression of ncRNAs in AS T cells, nine studies were included in the review. First, we briefly introduced the current theory for the pathogenesis of AS, especially the recent advance in T cells. Then, we introduced the ncRNAs and its overview in patients with AS. Finally, we summarized the results of the selected articles for the aberrant expression of ncRNAs in T cells from patients with AS and their roles in the immunopathogenesis of AS.

## 2. Pathogenesis of AS

AS was diagnosed according to the traditional modified New York criteria [9] or recent Assessment of SpondyloArthritis International Society classification criteria [10]. Disease activity was measured by the Bath Ankylosing Spondylitis Disease Activity Index [11] or Ankylosing Spondylitis Disease Activity Score (ASDAS) [12].

AS was once considered an autoinflammatory disease rather than autoimmune disease [13]. Autoinflammatory diseases are characterized by dysregulated self-direct inflammation caused by the activation of the innate immune system, and autoantibodies or autoantigen-specific T and B cells are absent. In contrast, autoimmune diseases are regarded as defects in either B or T lymphocyte selection, with aberrant adaptive immune responses to autoantigens [14]. However, current studies suggest that adaptive immunity, especially T cells, plays an important role in the pathogenesis of AS. We will briefly review the current theories, including genetic inheritance, environment factors, and T cells, regarding the pathogenesis of AS.

### 2.1. Genetic Inheritance

It is well known that around 85% of patients with AS are positive for HLA-B27, but only about 5% of HLA-B27-positive individuals develop AS [15]. HLA-B27 is not only a diagnostic marker for AS, but it also plays a critical role in the pathogenesis of AS [3]. There are three molecular mechanisms explaining the role of HLA-B27 in the pathogenesis of AS: (1) HLA-B27 misfolding, (2) formation of HLA-B27 heavy chain homodimers, and (3) presentation of arthritogenic peptides [16].

First, HLA-B27 tends to misfold, leading to endoplasmic reticulum stress and resulting in the unfolded protein response. In the HLA-B27 transgenic rat model, the unfolded protein response leads to increased secretion of interleukin (IL)-23 and thus IL-17 production by T helper 17 (Th17) lymphocytes [17]. Correcting the misfolded HLA-B27 would increase the membrane expression of HLA-B27 and the susceptibility of cell death mediated by cluster of differentiation (CD)8+ T-cell cytotoxicity [18] and the IL-23/IL-17 expression in PBMCs isolated from AS patients [19].

Second, two free heavy chains of HLA-B27 could form homodimer, which can stimulate innate and adaptive immune responses. The cell surface HLA-B27 homodimers could interact with the killer cell immunoglobulin (Ig)-like receptors and Ig-like transcripts expressed on CD4+ and CD8+ T cells and natural killer (NK) cells. Bowness et al. demonstrated that the interaction with HLA-B27 homodimers facilitated Th17 cells’ survival and proliferation [20].

The third hypothesis is that arthritogenic peptides derived from microbes are presented by HLA-B27 to stimulate CD8+ T cells, which subsequently interact with HLA-B27-bound self-peptides. After encountering with an infectious antigen, the HLA-B27 molecule could present an arthritogenic self-antigen through a well-known mechanism, molecular mimicry. Molecular mimicry interacts with an autoreactive CD8+ T lymphocytes, which would then trigger the inflammatory mechanisms leading to the disease [7].

In addition to the HLA-B27 gene, genetic studies also showed that genes involved in cytokine production, specifically genes in the Th17 pathway (*IL*-*23A*, *IL*-*12B*, and *IL*-*23R*), genes in the Th2 pathway (*IL*-*4* and *IL*-*13*), and genes in the nuclear factor κB (NFκB) pathway, contribute to the development of AS [21].

### 2.2. Environmental Factors

Recently, there have been significant advances in understanding the environmental factors contributing to the development of AS [22]. Since patients with AS have an elevated risk for inflammatory bowel disease [23], it is not surprising that gut dysbiosis is well known in patients with AS [24]. Costello et al. showed that there is a higher abundance of five families of bacteria, including Lachnospiraceae, Ruminococcaceae, Rikenellaceae, Porphyromonadaceae, and Bacteroidaceae, and a decrease in the abundance of two families of bacteria, Veillonellaceae and Prevotellaceae, at the terminal ileum microbial communities in patients with AS [25]. In addition, pathogens other than the gut microbiota, including COVID19, can increase the risk of developing AS [26,27]. Another important advancement in the environmental factors of AS is biomechanical stress. Patients with AS frequently developed enthesis, which is inflammation in the area where tendons or ligaments attach to bones. Jacques et al. showed that mechanical strain could cause enthesis and subsequent new bone formation in SpA [28].

### 2.3. T Cells

#### 2.3.1. CD4+ T Cells

In recent years, there have been major advances in our understanding of the role of T cells in the immunopathogenesis of AS. T cells can be divided into helper T (Th) cells and cytotoxic T (Tc) cells, also known as CD4+ T cells and CD8+ T cells, respectively. Th cells can be further classified into several subsets: Th1, Th2, Th17, regulatory T cells (Treg), T helper type 22 (Th22), and T follicular helper (Tfh) cells. These subsets of T helper cells produce characteristic cytokines: for example, interferon-gamma (IFN-γ) for Th1; IL-4, IL-5 and IL-13 for Th2, IL-17 for Th17, IL-10 and TGF-β for Treg; IL-22 for Th22 and IL-21 for Tfh.

Early studies showed the presence of T cells in the peripheral and sacroiliac joints of patients with AS, associating them with disease activity and response to treatment [29,30]. In earlier studies, T cells from patients with AS had elevated cell count and cytokines of Th1 cells compared to those of Th2 cells [31,32,33]. Chen et al. demonstrated that AS patients had higher serum IL-17 and IL-23 levels and their serum concentration correlated to disease activity measured by BASDAI scores [34]. In a meta-analysis, the proportion of Th17 cells and Th17-related cytokines (IL-17, IL-21, and IL-23) is elevated in patients with AS and even higher in those with active disease [35]. The most important finding is that using monoclonal antibody that selectively targets interleukin-17A has been approved for treating patients with active AS [36]. Contrary to Th17 cells, patients with AS have a lower proportion of Treg cells [37], as expected. However, Liao et al. showed that the frequency of Treg was positively correlated with the serum inflammatory marker CRP and ESR in patients with AS and decreased after effective treatment for AS. The condition for Treg is much more complex in AS patients with insufficient function of Treg cells [38]. In 2024, a review by Rodolfi et al. summarized the functional defects of Treg cells and their role in the pathogenesis of AS [39].

Recently, Th22, and Tfh cells have been identified. An increased frequency of Th22 cells and elevated serum levels of IL-22 have been detected in patients with AS, but whether these changes correlate with clinical activity in AS still needs further investigation [40,41]. In 2015, Shan et al. found a higher frequency of peripheral blood Tfh cells and elevated concentrations of their representative cytokines, serum IL-21, in patients with AS [42]. The main function of Tfh cells is to help B cells produce antibodies against pathogens. However, no known autoantibodies were involved in the pathogenesis of AS at that time.

#### 2.3.2. Formation of Autoantibodies

Posttranslational modifications (PTMs) of protein, such as phosphorylation, glycosylation, and citrullination of proteins are important mechanisms that change protein structure and function. PTMs also generate neoantigens and induce autoimmune reaction. In 2023, Zhai et al. surveyed a panel of peptides with PTM by mass spectrometry analysis in peripheral blood mononuclear cells (PBMCs) from patients with AS compared with the healthy control. They found that carboxyethylated integrin αIIb (ceITGA2B) induces autoantibody production and T-cell response in patients with AS, and anti–ceITGA2B antibodies titer being higher in the plasma of patients with AS compared to controls [43]. 3-hydroxypropionic acid (3-HPA), a metabolite commonly released from gut microbes, could promote protein cysteine carboxyethylation. The presence of autoantibodies in patients with AS opens a new field in AS research, deserving further studies.

#### 2.3.3. CD8+ T Cells

For the cytotoxic T cells, antigen presentation through HLA-B27 allows the stimulation of peptide-specific CD8+ T cells. However, few studies have investigated the potential CD8+ dysfunction in patients with AS. An early study showed that a higher proportion of IL4+ CD8+ cells in the peripheral blood of patients with AS [44], and these cells could produce tumor necrosis factor (TNF)-α, a key cytokine in the pathogenesis of AS [45]. Gracey et al. showed that CD8+ T cell frequency was reduced in the blood but increased in the synovial fluid of patients with AS [46]. Martini et al. showed that the phenotype of CD8+ CCR4+ T cells in active AS was altered, showing a decreased frequency of effector memory cells and an increased frequency of effector memory cells re-expressing CD45RA (TEMRA) T cells. TEMRA cells are terminally differentiated cells that have high cytotoxicity and play an important role in the pathogenesis of several autoimmune diseases [47]. This alternation was associated with the disease activity of AS. CD8+ CCR4+ T cells in AS exhibit increased secretion of perforin and granzyme B, which are key mediators for cytotoxicity. Also, several genes, including BMP receptors (*ACVR2A*, *ACVR2B*, *BMPR1A*, and *BMPR1B*), nephronectin (*NPNT*), insulin growth factor 1 (*IGF1*), and wingless-type MMTV integration site family member 5 (*WNT5*), which are involved in the positive regulation of ossification and osteoblast differentiation, are upregulated in CD8+ CCR4+ AS T cells [48].

## 3. NcRNAs

NcRNAs are RNA transcripts that do not translate into proteins and can be divided into housekeeping ncRNAs (such as transfer RNA (tRNA) and ribosomal RNA (rRNA)) and regulatory ncRNAs. The regulatory ncRNAs can be further classified into short ncRNAs (sncRNAs) or long ncRNAs (lncRNAs), containing less than or more than 200 nucleotides, respectively. SncRNAs mainly include microRNAs (miRNAs), PIWI-interacting RNAs (piRNAs), small nucleolar RNAs (snoRNAs), and small interfering RNAs (siRNAs). MiRNAs can repress translational messenger RNA (mRNA) [49] and have received the most attention in the sncRNA family. The classification of lncRNAs is much more complex. Traditionally, lncRNAs are classified into five broad categories: sense, antisense, bidirectional, intronic, and intergenic [50], and they modulate gene expression in a more complex way [50]. Recently, circular RNAs, generated by the back-splicing of transcripts, have also been included in the family of lncRNAs.

It is well known that the abnormal expression of ncRNAs in T cells from patients with rheumatoid arthritis (RA) or systemic lupus erythematosus (SLE) plays important roles, including altered gene transcription, cell signaling abnormalities, T-cell subpopulation alteration, aberrant cytokines and chemokines release, and abnormal activation of T cells, all of which contribute to their respective immunopathogenesis in SLE or RA [51]. Aberrantly expressed ncRNAs in patients with AS could regulate osteoblast proliferation, inflammatory response, new bone formation and T cell differentiation, and biological function [52]. In this review, we summarized the aberrant expression of ncRNAs in T cells from patients with AS and their known roles in the immunopathogenesis of AS.

### 3.1. Overview of Abnormal Expression of ncRNA in Patients with AS

AS is a chronic systemic autoimmune disease and differentially expressed ncRNAs from patients with AS have been identified in hip joint ligaments, peripheral blood, plasma, PBMCs, and osteogenically differentiated mesenchymal stem cells (MSCs). These ncRNAs can participate in new bone formation and inflammation. Clinically, the expression levels of miR-146a, miR-125a-5p, miR-125b-5p, miR-499a, and miR-155a from whole blood and the expression levels of hsa_circRNA_001544 from PBMCs are mentioned as potential biomarkers for the diagnosis of AS. Whole blood miR-125a-5p and miR-155a expression levels and PBMC hsa_circRNA_012732 expression have been purposed as potential indicators of AS disease activity [53,54]. Li et al. showed that the expression levels of lncRNA intersectin 1-2 in AS PBMCs declined during TNFα inhibitor treatment and correlated with a good treatment response in patients with AS [55]. These ncRNAs have been reviewed in more detail in other articles [56,57].

In this review, we focus on the differentially expressed ncRNAs in T cells from patients with AS and their roles in the pathogenesis of AS. We searched PubMed and used the terms: “ankylosing spondylitis or axial spondyloarthritis”, “ncRNAs”, and “T cells” for studies published between January 2013 and June 2024. Twenty papers were found, and after excluding review articles and those not relevant to the expression of ncRNAs in AS T cells, nine studies were included in the review.

### 3.2. Abnormal Expression of ncRNAs in AS T Cells

In 2013, Lai et al. demonstrated that abnormal expression of ncRNAs participated in the immunopathogenesis of AS [58]. The expression levels of miR-16, miR-221, and let-7i were increased in T cells from 22 patients with AS compared to 18 healthy controls, and the expression of miR-221 and let-7i positively correlated with radiographic changes in the lumbar spine of patients with AS. Later, Reyes-Loyola et al. showed that serum levels of let-7i were higher in 15 patients with AS than in 13 controls [59]. We confirmed that Toll-like receptor-4 (TLR-4) is a target of let-7i cells from patients with AS. Hou et al. provided another molecular mechanism. Autophagy is a self-degradative mechanism in response to nutrient deficiency. Autophagy can remove misfolded or aggregated proteins, clear damaged organelles, such as mitochondria, peroxisomes, and endoplasmic reticulum as well as eliminate intracellular pathogens [60]. They found that overexpression of let-7i in Jurkat cells significantly suppressed insulin-like growth factor 1 receptor expression and that impaired IGF signaling is an important way of inducing autophagy. This led to the decreased phosphorylation of mammalian targets of rapamycin (mTOR) and Akt, downregulation of Bcl-2, upregulation of Bax, and cleavage of caspase 3 and polyadenosine diphosphate-ribose polymerase. Let-7i overexpression induced autophagy, which helped protect cells from apoptosis [61]. Increased expression of let-7i enhanced interferon (IFN)-γ production in activated T cells, contributing to an enhanced Th1 response in AS. However, one study showed different results in 10 patients with AS and 10 controls [62]. Due to the small sample size of these studies, further research with a larger sample is needed.

In 2017, Wang et al. showed that the expression levels of miR-199a-5p were down-regulated in T cells from patients with AS [63]. Overexpression of miR-199a-5p promoted the protein levels of autophagy-related genes, including rat microtubule-associated protein 1 light chain 3 (LC3)-II, beclin1, and ATG5 in both Jurkat cells and T cells from patients with AS. This finding supports the idea that patients with AS have decreased expression of genes associated with autophagy. The extent of the reduction in autophagy-related gene 5 (ATG5) and ATG12 expression levels correlated with disease severity and activity in patients with AS [64]. Overexpression of miR-199a-5p also decreased TNF-α, IL-17, and IL-23 expression in both Jurkat cells and AS T cells. Ras Homolog Enriched in Brain (Rheb) is confirmed to be the target of miR-199a-5p. Overexpression of Rheb suppressed T cell autophagy and promoted pro-inflammatory cytokine production by activating mTOR signaling, participating in the inflammatory response of AS.

Since Th17 cells play an important role in the immunopathogenesis of AS, Chen et al. found that expression levels miR-10b-5p, miR-210-3p, and miR-155-5p were elevated in Th17 cells compared with non-Th17 cells in patients with AS. Enhanced expression of miR-10b-5p reduced the frequency Th17 cells and IL-17A production in CD4+ T cells by targeting mitogen-activated protein 3 kinase 7 (MAP3K7). The addition of IL-6 and TNF-α upregulated the expression of miR-10b-5p, which in turn suppressed IL-17A production [65]. IL-23, mainly secreted by activated macrophages and dendritic cells (DCs), is an important cytokine that induces the differentiation of Th17 cells [66]. We speculated that IL-23 could regulate the expression of miRNAs in T cells from patients with AS, and these IL-23-regulated miRNAs could contribute to the immunopathogenesis of AS. We found that in the IL-23-regulated miRNAs, the higher expression levels of miR-29b-1-5p, miR-211-3p, miR-4449, miR-7114-5p, and miR-1914-3p were identified in T cells from patients with AS. Overexpression of miR-29b-1-5p inhibited IL-23-mediated signal transducer and activator of transcription 3 (STAT3) phosphorylation. Overexpression of miR-29b-1-5p or miR-211-3p increased IFN-γ expression [67]. However, blocking IL-23 failed to show any evidence of clinical efficacy for patients with AS in clinical studies [68]. Two hypotheses attempt to explain this result. The first is that IL-23 contributes to disease initiation but becomes redundant in established diseases. Second, IL-17 is not only produced by canonical Th17 cells but also by different cells, including gamma delta T cells, mucosal-associated invariant T cells, invariant natural killer cells, and type 3 innate lymphoid cells, which are less dependent on IL-23 for their IL-17 production [69]. Clearly, more studies are needed to clarify this critical issue.

Fogel et al. studied T cells and monocytes from 81 patients with AS, fulfilling the 2009 ASAS classification criteria and 55 controls. They found that miR-16-1-3p, miR-28-5p, miR-199a-5p, miR-126-3p, let-7d-3p, miR-484, miR-16-5p, and miR197-3p were upregulated, while miR-361-3p, miR-181c-5p, and miR-874-3p were downregulated in AS T cells compared with the controls [70]. Bioinformatic analysis was used to identify the potential related molecular pathways, but no further analysis was provided.

Li et al. showed that the expression of miR-130a-3p was lower in T cells from patients with AS. Inhibition of miR-130a-3p significantly inhibited cell proliferation and induced cell apoptosis in Jurkat cells by targeting homeobox B1 [71]. However, the role of T cell reduction in the pathogenesis of AS remains unclear.

Yu et al. demonstrated that the expression of lncRNA LOC645166 is lower in T cells from patients with AS compared with controls [72]. Overexpression of LOC645166 down-regulated the IL-23 expression and suppressed the JAK2/STAT3 signaling pathway. The JAK/STAT signaling pathway, activated by cytokine/cytokine receptor systems and growth factors, is well known for its role in immune response, T-cell proliferation, and T-cell apoptosis [73]. This pathway is known to participate in the pathogenesis of multiple autoimmune conditions, including AS. The JAK/STAT pathway participated in the pathogenesis of AS through affecting Treg development, facilitating T-cell survival and activation and inducing Th1/Th17 differentiation [73]. JAK inhibitors are now proven to be a novel, effective treatment for AS, making the JAK/STAT pathway a new and important issue for T-cell dysfunction in AS [74]. We found that LOC645166 binds to K63-linked polyubiquitin chains and can suppress the recruitment of the IkappaB kinase (IKK) complex to K63-linked polyubiquitin chains, and suppress IKK2 activation. This leads to the decreased phosphorylation of IkB and nuclear translocation of p50. Decreased expression of LOC645166 contributes to an inflammatory response in AS.

**Figure 1:**
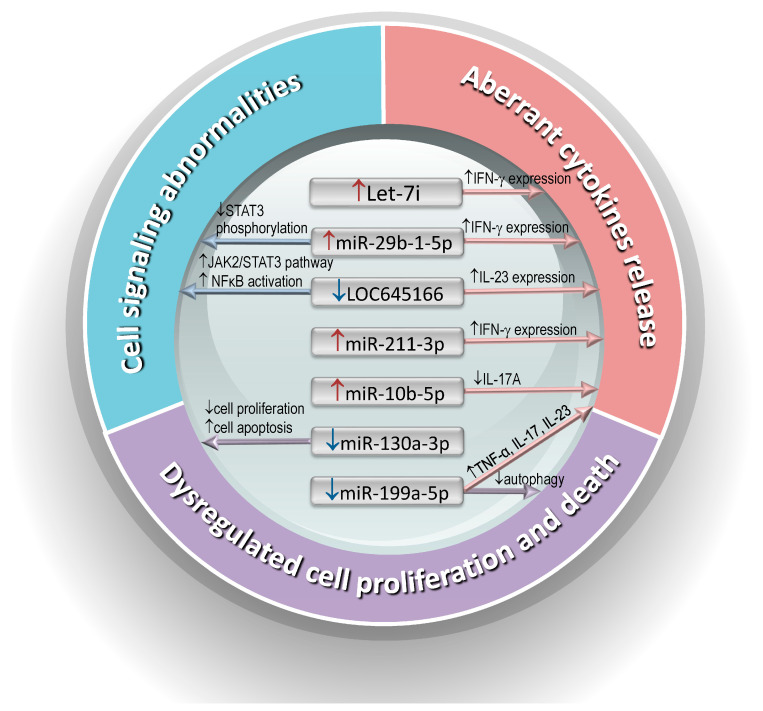
The aberrantly expressed ncRNAs involved in aberrant cytokine release, dysregulated cell proliferation and death, and cell signaling abnormalities in T cells from patients with ankylosing spondylitis contribute to its immunopathogenesis.

According to current studies, aberrantly expressed ncRNAs in AS T cells can cause abnormal cytokine release, cell signaling abnormalities, and dysregulated cell proliferation and death, all of which contribute to the immunopathogenesis of AS (Figure 1).

In addition to the endogenous aberrantly expressed ncRNAs in T cells from patients with AS affecting T-cell function, exogenous ncRNAs can also affect T cell function in patients with AS. Exosomes are small extracellular vesicles containing proteins, mRNAs, and ncRNAs, which are important for intercellular communication. Exosomes are produced by a variety of cells and have been identified in body fluid, including plasma. Tavasolian et al. showed that exosomes purified from patients with AS could inhibit the proliferation of FOXP3+ Treg cells, decrease the frequency of FOXP3+IRF4+ Treg cells, and decrease the secretion of IL-8 and IL-10 in healthy T cells [74]. These effects could be mediated by miRNA.

We summarized the results from nine studies of non-coding RNAs associated with T-cell dysfunction in patients with AS in Table 1.

## 4. Discussion

Based on the current studies, abnormal ncRNA expression in T cells participates in abnormal cytokine release, cell signaling abnormalities, and dysregulated cell proliferation and death in AS T cells. We noted that the number of studies investigating the functional roles of abnormal expression ncRNAs in AS T cells are still very low compared to those in RA or SLE. Moreover, the expression profile of some components of ncRNAs, such as circular RNAs, is not currently available in AS T cells.

Several research fields need exploration. First, the imbalance in Th17 and Treg cells is well documented in patients with AS, and ncRNAs are known to regulate the differentiation of T cells [76]. Therefore, it is anticipated that some ncRNAs would participate in the dysregulation of T cell differentiation in patients with AS. Second, abnormal T cell metabolism also plays a critical role in the pathogenesis of autoimmune diseases. Few studies have addressed the abnormal metabolism in AS T cells and the role of ncRNAs in it. Since HLA-B27 plays a critical role in the immunopathogenesis of AS, including misfolding and unfolded protein response (UPR), it is important to explore whether HLA-B27 itself is related to abnormal ncRNA expression or of ncRNAs participating in the process of misfolding and UPR. Finally, gut microbiota also participate in the pathogenesis of AS. Gut microbiota can affect the ncRNA expression in the intestine and the immune system [77]. Moreover, gut microbiota could communicate with distant organs such as the brain via microbiota-derived extracellular vesicles [78]. The interaction of ncRNAs and dysregulated microbiota for the pathogenesis of AS is an interesting topic that deserves additional research.

## 5. Conclusions

In the past decades, there has been major advances in the immunopathogenesis of AS and adaptive immunity has become a key factor. Moreover, research studies for the abnormal ncRNAs in AS, either for diagnosis, associated with clinical responses, or pathogenesis, are rapidly growing. In this review, we summarized the up-to-date studies investigating abnormally expressed ncRNAs in AS T cells, focusing on those detailing molecular mechanisms. Our review showed that abnormal ncRNA expression in T cells leads to abnormal cytokine release, cell signaling abnormalities, and dysregulated cell proliferation and death, which contribute to the inflammatory response of AS. We anticipated that more aberrantly expressed ncRNAs will be discovered in AS T cells, further contributing to the domain of T-cell dysfunction in AS.

## Figures and Tables

**Table 1:** Summary for the known non-coding RNAs associated with T cells dysfunction in patients with ankylosing spondylitis.

ncRNAs	Validated Targets	Signaling Pathway	Function	Patient Number	Reference and Study Year
miR-16↑, miR-221↑and let-7i↑	TLR-4	LPS/TLR-4	Enhanced IFN-γ expression	22	2013 [58]
let-7i	IGF-1R	mTOR/AKT	Induced autophagy	-	2014 [61]
miR-199a-5p↓	Rheb	mTOR	Inhibited autophagy	41	2017 [63]
miR-10b-5p↑, miR-155-5p↑, and miR-210-3p↑	MAP3K7	MAPKs	Reduced Th17 differentiation and IL-17A production	15	2017 [65]
miR-16-1-3p↑, miR-28-5p↑, miR-199a-5p↑, and miR-126-3p↑	-	-	-	81	2019 [70]
miR-130a-3p↓	Homeobox B1	Bcl-2/Bax pathway	Inhibited T-cell proliferation and induced T-cell apoptosis	30	2019 [71]
miR-29b-1-5p↑, miR-4449↑, miR-211-3p↑, miR-1914-3p↑, and miR-7114-5p↑	Angiogenin	JAK/STAT pathway	Enhanced IFN-γ expression	24	2018 [67]
LOC100506014↓, LOC645166↓, lncEXD2-1↓, and LINC00282↓	K63-linked polyubiquitin chains	JAK/STAT and NFkB pathway	Enhanced IL-23 expression	30	2021 [72]
* let-7b-5p↓ and let-7c-5p↓ miR-30d-5p↑, miR-93-5p↑, miR-23b-3p↑, miR-191–5p↑, miR-148b-3p↑,miR-146b-5p↑,miR-1260b↑, miR-130a-5p↑, miR-140-3p↑, miR-145-3p↑, miR-22-5p↑, miR-2277-5p↑, miR-27a-3p↑, miR-29a-3p↑, miR-30c-5p↑, miR-330-5p↑, miR-345-5p↑, miR-4717-3p↑, miR-500a-3p↑, miR-502-3p↑, miR-598-3p↑, miR-6810-3p↑	-	Covalent chromatin modification, chromatin modifying enzymes, nuclear chromatin, chromatin DNA binding	Inhibited the proliferation of regulatory T cells	17	2023 [75]

TLR-4: Toll-like receptor 4; LPS: Lipopolysaccharide; IFN-γ: interferon-γ; IGF-1R: insulin-like growth factor 1 receptor; mTOR: mammalian target of rapamycin; AKT: protein kinase B; Rheb: Ras Homolog Enriched in Brain; MAP3K7: mitogen-activated protein kinase kinase kinase 7; MAPKs: mitogen-activated protein kinases; B cell lymphoma 2 (Bcl-2); Bax: Bcl-2–associated X protein; JAK: Janus kinase; STAT: signal transducer and activator of transcription; NFkB: nuclear factor kappa-light-chain enhancer of activated B cells. * The expression of ncRNAs in exosomes from patients with AS.

## References

[1] van der Heijde D., Molto A., Ramiro S., Braun J., Dougados M., van Gaalen F.A., Gensler L.S., Inman R.D., Landewé R.B.M., Marzo-Ortega H. (2024). Goodbye to the term ‘ankylosing spondylitis’, hello ‘axial spondyloarthritis’: Time to embrace the ASAS-defined nomenclature. Ann. Rheum. Dis..

[2] Taurog J.D., Chhabra A., Colbert R.A. (2016). Ankylosing Spondylitis and Axial Spondyloarthritis. N. Engl. J. Med..

[3] Sieper J., Poddubnyy D. (2017). Axial spondyloarthritis. Lancet.

[4] Chaudhary H., Bohra N., Syed K., Donato A., Murad M.H., Karmacharya P. (2023). All-cause and cause-specific mortality in psoriatic arthritis and ankylosing spondylitis: A systematic review and meta-analysis. Arthritis Care Res..

[5] Schlosstein L., Terasaki P.I., Bluestone R., Pearson C.M. (1973). High association of an HL-A antigen, W27, with ankylosing spondylitis. N. Engl. J. Med..

[6] Ganau L., Prisco L., Ligarotti G.K.I., Ambu R., Ganau M. (2018). Understanding the pathological basis of neurological diseases through diagnostic platforms based on innovations in biomedical engineering: New concepts and theranostics perspectives. Medicines.

[7] Rosine N., Fogel O., Koturan S., Rogge L., Bianchi E., Miceli-Richard C. (2023). T cells in the pathogenesis of axial spondyloarthritis. Jt. Bone Spine.

[8] Wang Z., Chang C., Lu Q. (2017). Epigenetics of CD4^+^ T cells in autoimmune diseases. Curr. Opin. Rheumatol..

[9] van der Linden S., Valkenburg H.A., Cats A. (1984). Evaluation of diagnostic criteria for ankylosing spondylitis. Arthritis Rheum..

[10] Rudwaleit M., van der Heijde D., Landewé R., Akkoc N., Brandt J., Chou C.T., Dougados M., Huang F., Gu J., Kirazli Y. (2011). The Assessment of SpondyloArthritis International Society classification criteria for peripheral spondyloarthritis and for spondyloarthritis in general. Ann. Rheum. Dis..

[11] Garrett S., Jenkinson T., Kennedy L.G., Whitelock H., Gaisford P., Calin A. (1994). A new approach to defining disease status in ankylosing spondylitis: The Bath Ankylosing Spondylitis Disease Activity Index. J. Rheumatol..

[12] Machado P., Landewé R., Lie E., Kvien T.K., Braun J., Baker D., van der Heijde D. (2011). Ankylosing Spondylitis Disease Activity Score (ASDAS): Defining cut-off values for disease activity states and improvement scores. Ann. Rheum. Dis..

[13] Ambarus C., Yeremenko N., Tak P.P., Baeten D. (2012). Pathogenesis of spondyloarthritis: Autoimmune or autoinflammatory?. Curr. Opin. Rheumatol..

[14] McGonagle D., McDermott M.F. (2006). A proposed classification of the immunological diseases. PLoS Med..

[15] Braun J., Bollow M., Remlinger G., Eggens U., Rudwaleit M., Distler A., Sieper J. (1998). Prevalence of spondylarthropathies in HLA-B27 positive and negative blood donors. Arthritis Rheum..

[16] Braun J., Sieper J. (2023). Fifty years after the discovery of the association of HLA B27 with ankylosing spondylitis. RMD Open.

[17] DeLay M.L., Turner M.J., Klenk E.I., Smith J.A., Sowders D.P., Colbert R.A. (2009). HLA-B27 misfolding and the unfolded protein response augment interleukin-23 production and are associated with Th17 activation in transgenic rats. Arthritis Rheum..

[18] Yu H.C., Lu M.C., Li C., Huang H.L., Huang K.Y., Liu S.Q., Lai N.S., Huang H.B. (2013). Targeted delivery of an antigenic peptide to the endoplasmic reticulum: Application for development of a peptide therapy for ankylosing spondylitis. PLoS ONE.

[19] Yu H.C., Huang K.Y., Lu M.C., Huang H.L., Liu S.Q., Lai N.S., Huang H.B. (2017). Targeted Delivery of the HLA-B^∗^27-Binding Peptide into the Endoplasmic Reticulum Suppresses the IL-23/IL-17 Axis of Immune Cells in Spondylarthritis. Mediat. Inflamm..

[20] Bowness P., Ridley A., Shaw J., Chan A.T., Wong-Baeza I., Fleming M., Cummings F., McMichael A., Kollnberger S. (2011). Th17 cells expressing KIR3DL2^+^ and responsive to HLA-B27 homodimers are increased in ankylosing spondylitis. J. Immunol..

[21] Reveille J.D. (2011). The genetic basis of spondyloarthritis. Ann. Rheum. Dis..

[22] Simone D., Al Mossawi M.H., Bowness P. (2018). Progress in our understanding of the pathogenesis of ankylosing spondylitis. Rheumatology.

[23] Rudwaleit M., Baeten D. (2006). Ankylosing spondylitis and bowel disease. Best Pract. Research. Clin. Rheumatol..

[24] Song Z.Y., Yuan D., Zhang S.X. (2022). Role of the microbiome and its metabolites in ankylosing spondylitis. Front. Immunol..

[25] Costello M.E., Ciccia F., Willner D., Warrington N., Robinson P.C., Gardiner B., Marshall M., Kenna T.J., Triolo G., Brown M.A. (2015). Brief Report: Intestinal Dysbiosis in Ankylosing Spondylitis. Arthritis Rheumatol..

[26] Kim S.H., Lee S.H. (2023). Updates on ankylosing spondylitis: Pathogenesis and therapeutic agents. J. Rheum. Dis..

[27] Chang R., Yen-Ting Chen T., Wang S.I., Hung Y.M., Chen H.Y., Wei C.J. (2023). Risk of autoimmune diseases in patients with COVID-19: A retrospective cohort study. EClinicalMedicine.

[28] Jacques P., Lambrecht S., Verheugen E., Pauwels E., Kollias G., Armaka M., Verhoye M., Van der Linden A., Achten R., Lories R.J. (2014). Proof of concept: Enthesitis and new bone formation in spondyloarthritis are driven by mechanical strain and stromal cells. Ann. Rheum. Dis..

[29] Baeten D., Kruithof E., Van den Bosch F., Demetter P., Van Damme N., Cuvelier C., De Vos M., Mielants H., Veys E.M., De Keyser F. (2001). Immunomodulatory effects of anti-tumor necrosis factor alpha therapy on synovium in spondylarthropathy: Histologic findings in eight patients from an open-label pilot study. Arthritis Rheum..

[30] Bollow M., Fischer T., Reisshauer H., Backhaus M., Sieper J., Hamm B., Braun J. (2000). Quantitative analyses of sacroiliac biopsies in spondyloarthropathies: T cells and macrophages predominate in early and active sacroiliitis—Cellularity correlates with the degree of enhancement detected by magnetic resonance imaging. Ann. Rheum. Dis..

[31] Wen J.T., Zhang D.H., Fang P.F., Li M.H., Wang R.J., Li S.H. (2017). Role of Th1/Th2 cytokines in the diagnosis and prognostic evaluation of ankylosing spondylitis. Genet. Mol. Res..

[32] Roberts A.R., Vecellio M., Chen L., Ridley A., Cortes A., Knight J.C., Bowness P., Cohen C.J., Wordsworth B.P. (2016). An ankylosing spondylitis-associated genetic variant in the IL23R-IL12RB2 intergenic region modulates enhancer activity and is associated with increased Th1-cell differentiation. Ann. Rheum. Dis..

[33] Shesternya P.A., Savchenko A.A., Gritsenko O.D., Vasileva A.O., Kudryavtsev I.V., Masterova A.A., Isakov D.V., Borisov A.G. (2022). Features of peripheral blood th-cell subset composition and serum cytokine level in patients with activity-driven ankylosing spondylitis. Pharmaceuticals.

[34] Chen W.S., Chang Y.S., Lin K.C., Lai C.C., Wang S.H., Hsiao K.H., Lee H.T., Chen M.H., Tsai C.Y., Chou C.T. (2012). Association of serum interleukin-17 and interleukin-23 levels with disease activity in Chinese patients with ankylosing spondylitis. J. Chin. Med. Assoc..

[35] Su Q.Y., Zheng J.W., Yang J.Y., Zhang T.Y., Song S., Zhao R., Di J.K., Zhang S.X., Wang C.H., Gao H.Y. (2022). Levels of peripheral th17 cells and th17-related cytokines in patients with ankylosing spondylitis: A meta-analysis. Adv. Ther..

[36] van der Heijde D., Cheng-Chung Wei J., Dougados M., Mease P., Deodhar A., Maksymowych W.P., Van den Bosch F., Sieper J., Tomita T., Landewé R. (2018). Ixekizumab, an interleukin-17A antagonist in the treatment of ankylosing spondylitis or radiographic axial spondyloarthritis in patients previously untreated with biological disease-modifying anti-rheumatic drugs (COAST-V): 16 week results of a phase 3 randomised, double-blind, active-controlled and placebo-controlled trial. Lancet.

[37] Lai N.L., Zhang S.X., Wang J., Zhang J.Q., Wang C.H., Gao C., Li X.F. (2019). The Proportion of Regulatory T Cells in Patients with Ankylosing Spondylitis: A Meta-Analysis. J. Immunol. Res..

[38] Liao H.T., Tsai C.Y. (2023). Cytokines and regulatory T cells in ankylosing spondylitis. Bone Jt. Res..

[39] Rodolfi S., Davidson C., Vecellio M. (2023). Regulatory T cells in spondyloarthropathies: Genetic evidence, functional role, and therapeutic possibilities. Front. Immunol..

[40] Lejon K., Hellman U., Do L., Kumar A., Forsblad-d’Elia H. (2022). Increased proportions of inflammatory T cells and their correlations with cytokines and clinical parameters in patients with ankylosing spondylitis from northern Sweden. Scand. J. Immunol..

[41] Zhang L., Li Y.G., Li Y.H., Qi L., Liu X.G., Yuan C.Z., Hu N.W., Ma D.X., Li Z.F., Yang Q. (2012). Increased frequencies of Th22 cells as well as Th17 cells in the peripheral blood of patients with ankylosing spondylitis and rheumatoid arthritis. PLoS ONE.

[42] Xiao F., Zhang H.Y., Liu Y.J., Zhao D., Shan Y.X., Jiang Y.F. (2013). Higher frequency of peripheral blood interleukin 21 positive follicular helper T cells in patients with ankylosing spondylitis. J. Rheumatol..

[43] Zhai Y., Chen L., Zhao Q., Zheng Z.H., Chen Z.N., Bian H., Yang X., Lu H.Y., Lin P., Chen X. (2023). Cysteine carboxyethylation generates neoantigens to induce HLA-restricted autoimmunity. Science.

[44] Zhang L., Jarvis L.B., Baek H.J., Gaston J.S. (2009). Regulatory IL4+CD8+ T cells in patients with ankylosing spondylitis and healthy controls. Ann. Rheum. Dis..

[45] Baek H.J., Zhang L., Jarvis L.B., Gaston J.S. (2008). Increased IL-4+ CD8+ T cells in peripheral blood and autoreactive CD8+ T cell lines of patients with inflammatory arthritis. Rheumatology.

[46] Gracey E., Yao Y., Qaiyum Z., Lim M., Tang M., Inman R.D. (2020). Altered Cytotoxicity Profile of CD8+ T Cells in Ankylosing Spondylitis. Arthritis Rheumatol..

[47] Perl A., Morel L. (2023). Expanding scope of TEMRA in autoimmunity. EBioMedicine.

[48] Martini V., Silvestri Y., Ciurea A., Möller B., Danelon G., Flamigni F., Jarrossay D., Kwee I., Foglierini M., Rinaldi A. (2024). Patients with ankylosing spondylitis present a distinct CD8 T cell subset with osteogenic and cytotoxic potential. RMD Open.

[49] Lu M.C. (2023). Regulatory RNAs in rheumatology: From pathogenesis to potential therapy. Int. J. Rheum. Dis..

[50] Ponting C.P., Oliver P.L., Reik W. (2009). Evolution and functions of long noncoding RNAs. Cell.

[51] Lai N.S., Koo M., Yu C.L., Lu M.C. (2017). Immunopathogenesis of systemic lupus erythematosus and rheumatoid arthritis: The role of aberrant expression of non-coding RNAs in T cells. Clin. Exp. Immunol..

[52] Yang H., Chen Y., Xu W., Shao M., Deng J., Xu S., Gao X., Guan S., Wang J., Xu S. (2021). Epigenetics of ankylosing spondylitis: Recent developments. Int. J. Rheum. Dis..

[53] Tan H., Ren R., Zhang J., Huang Z., Niu Q., Yang B. (2022). Analysis of inflammation-related microRNA expression in patients with ankylosing spondylitis. Immunol. Res..

[54] Tang Y.P., Zhang Q.B., Dai F., Liao X., Dong Z.R., Yi T., Qing Y.F. (2021). Circular RNAs in peripheral blood mononuclear cells from ankylosing spondylitis. Chin. Med. J..

[55] Li M., Zhou X. (2021). Long noncoding RNA intersectin 1-2 gradually declines during adalimumab treatment, and its reduction correlates with treatment efficacy in patients with ankylosing spondylitis. Inflammopharmacology.

[56] Sun R., Wang X., Sun X., Zhao B., Zhang X., Gong X., Wong S.H., Chan M.T.V., Wu W.K.K. (2022). emerging roles of long non-coding RNAS in ankylosing spondylitis. Front. Immunol..

[57] Fang Y., Liu J. (2023). Novel regulatory role of non-coding RNAs in ankylosing spondylitis. Front. Immunol..

[58] Lai N.S., Yu H.C., Chen H.C., Yu C.L., Huang H.B., Lu M.C. (2013). Aberrant expression of microRNAs in T cells from patients with ankylosing spondylitis contributes to the immunopathogenesis. Clin. Exp. Immunol..

[59] Reyes-Loyola P., Rodríguez-Henríquez P., Ballinas-Verdugo M.A., Amezcua-Castillo L.M., Juárez-Vicuña Y., Jiménez-Rojas V., Márquez-Velasco R., Sánchez-Muñoz F., Amezcua-Guerra L.M. (2019). Plasma let-7i, miR-16, and miR-221 levels as candidate biomarkers for the assessment of ankylosing spondylitis in Mexican patients naïve to anti-TNF therapy. Clin. Rheumatol..

[60] Glick D., Barth S., Macleod K.F. (2010). Autophagy: Cellular and molecular mechanisms. J. Pathol..

[61] Hou C., Zhu M., Sun M., Lin Y. (2014). MicroRNA let-7i induced autophagy to protect T cell from apoptosis by targeting IGF1R. Biochem. Biophys. Res. Commun..

[62] Lu L., Fang H., Gu M., Wang H., Yu Q., Chen A., Gan K.F. (2023). MicroRNA Let-7i Regulates Innate TLR4 Pathways in Peripheral Blood Mononuclear Cells of Patients with Ankylosing Spondylitis. Int. J. Gen. Med..

[63] Wang Y., Luo J., Wang X., Yang B., Cui L. (2017). MicroRNA-199a-5p induced autophagy and inhibits the pathogenesis of ankylosing spondylitis by modulating the mTOR signaling via directly targeting Ras homolog enriched in brain (Rheb). Cell. Physiol. Biochem..

[64] Tan M., Zhang Q.B., Liu T.H., Yang Y.Y., Zheng J.X., Zhou W.J., Xiong Q., Qing Y.F. (2020). Autophagy dysfunction may be involved in the pathogenesis of ankylosing spondylitis. Exp. Ther. Med..

[65] Chen L., Al-Mossawi M.H., Ridley A., Sekine T., Hammitzsch A., de Wit J., Simone D., Shi H., Penkava F., Kurowska-Stolarska M. (2017). miR-10b-5p is a novel Th17 regulator present in Th17 cells from ankylosing spondylitis. Ann. Rheum. Dis..

[66] Schinocca C., Rizzo C., Fasano S., Grasso G., La Barbera L., Ciccia F., Guggino G. (2021). Role of the IL-23/IL-17 pathway in rheumatic diseases: An overview. Front. Immunol..

[67] Lai N.S., Yu H.C., Tung C.H., Huang K.Y., Huang H.B., Lu M.C. (2018). Aberrant expression of interleukin-23-regulated miRNAs in T cells from patients with ankylosing spondylitis. Arthritis Res. Ther..

[68] Baeten D., Østergaard M., Wei J.C., Sieper J., Järvinen P., Tam L.S., Salvarani C., Kim T.H., Solinger A., Datsenko Y. (2018). Risankizumab, an IL-23 inhibitor, for ankylosing spondylitis: Results of a randomised, double-blind, placebo-controlled, proof-of-concept, dose-finding phase 2 study. Ann. Rheum. Dis..

[69] Baeten D., Adamopoulos I.E. (2020). IL-23 Inhibition in ankylosing spondylitis: Where did it go wrong?. Front. Immunol..

[70] Fogel O., Bugge Tinggaard A., Fagny M., Sigrist N., Roche E., Leclere L., Deleuze J.F., Batteux F., Dougados M., Miceli-Richard C. (2019). Deregulation of microRNA expression in monocytes and CD4^+^ T lymphocytes from patients with axial spondyloarthritis. Arthritis Res. Ther..

[71] Li F., Si D., Guo X., Guo N., Li D., Zhang L., Jian X., Ma J. (2019). Aberrant expression of miR-130a-3p in ankylosing spondylitis and its role in regulating T-cell survival. Mol. Med. Rep..

[72] Yu H.C., Huang K.Y., Lu M.C., Huang Tseng H.Y., Liu S.Q., Lai N.S., Huang H.B. (2021). Down-regulation of LOC645166 in T cells of ankylosing spondylitis patients promotes the NF-κB signaling via decreasingly blocking recruitment of the IKK complex to K63-linked polyubiquitin chains. Front. Immunol..

[73] Raychaudhuri S.P., Shah R.J., Banerjee S., Raychaudhuri S.K. (2024). JAK-STAT signaling and beyond in the pathogenesis of spondyloarthritis and their clinical significance. Curr. Rheumatol. Rep..

[74] Deodhar A., Sliwinska-Stanczyk P., Xu H., Baraliakos X., Gensler L.S., Fleishaker D., Wang L., Wu J., Menon S., Wang C. (2021). Tofacitinib for the treatment of ankylosing spondylitis: A phase III, randomised, double-blind, placebo-controlled study. Ann. Rheum. Dis..

[75] Tavasolian F., Lively S., Pastrello C., Tang M., Lim M., Pacheco A., Qaiyum Z., Yau E., Baskurt Z., Jurisica I. (2023). Proteomic and genomic profiling of plasma exosomes from patients with ankylosing spondylitis. Ann. Rheum. Dis..

[76] Liu C., Yang H., Shi W., Wang T., Ruan Q. (2018). MicroRNA-mediated regulation of T helper type 17/regulatory T-cell balance in autoimmune disease. Immunology.

[77] Bi K., Zhang X., Chen W., Diao H. (2020). MicroRNAs regulate intestinal immunity and gut microbiota for gastrointestinal health: A comprehensive review. Genes.

[78] Cuesta C.M., Guerri C., Ureña J., Pascual M. (2021). Role of microbiota-derived extracellular vesicles in gut-brain communication. Int. J. Mol. Sci..

